# Systematic characterization of chromatin modifying enzymes identifies KDM3B as a critical regulator in castration resistant prostate cancer

**DOI:** 10.1038/s41388-019-1116-8

**Published:** 2019-12-10

**Authors:** Hilal Saraç, Tunç Morova, Elisabete Pires, James McCullagh, Anıl Kaplan, Ahmet Cingöz, Tugba Bagci-Onder, Tamer Önder, Akane Kawamura, Nathan A. Lack

**Affiliations:** 1 School of Medicine, Koç University, Istanbul, 34450 Turkey; 2 Chemistry Research Laboratory, Department of Chemistry, University of Oxford, Oxford, OX1 3TA UK; 3 Radcliffe Department of Medicine, Wellcome Centre for Human Genetics, University of Oxford, Oxford, OX3 7BN UK; 4 Vancouver Prostate Centre, University of British Columbia, Vancouver, V6H 3Z6 Canada

**Keywords:** Prostate cancer, Epigenetics, Histone post-translational modifications, RNAi

## Abstract

Androgen deprivation therapy (ADT) is the standard care for prostate cancer (PCa) patients who fail surgery or radiotherapy. While initially effective, the cancer almost always recurs as a more aggressive castration resistant prostate cancer (CRPC). Previous studies have demonstrated that chromatin modifying enzymes can play a critical role in the conversion to CRPC. However, only a handful of these potential pharmacological targets have been tested. Therefore, in this study, we conducted a focused shRNA screen of chromatin modifying enzymes previously shown to be involved in cellular differentiation. We found that altering the balance between histone methylation and demethylation impacted growth and proliferation. Of all genes tested, KDM3B, a histone H3K9 demethylase, was found to have the most antiproliferative effect. These results were phenocopied with a KDM3B CRISPR/Cas9 knockout. When tested in several PCa cell lines, the decrease in proliferation was remarkably specific to androgen-independent cells. Genetic rescue experiments showed that only the enzymatically active KDM3B could recover the phenotype. Surprisingly, despite the decreased proliferation of androgen-independent cell no alterations in the cell cycle distribution were observed following KDM3B knockdown. Whole transcriptome analyses revealed changes in the gene expression profile following loss of KDM3B, including downregulation of metabolic enzymes such as *ARG2* and *RDH11*. Metabolomic analysis of KDM3B knockout showed a decrease in several critical amino acids. Overall, our work reveals, for the first time, the specificity and the dependence of KDM3B in CRPC proliferation.

## Introduction

Androgen receptor (AR) signaling is critical at all stages of prostate cancer (PCa) progression. Given this essential role, recurrent, advanced, or metastatic PCa is commonly treated with androgen deprivation therapy (ADT) [1]. Typically, this treatment is initially successful and causes a decrease in both serum prostate specific antigen (PSA) and tumor volume [1, 2]. However, ADT is not curative and in almost all patients the cancer develops resistances and recurs as a more aggressive castration resistant prostate cancer (CRPC) [2, 3]. While there is almost no circulating androgen in these patients, extensive clinical evidence has demonstrated that the AR still remains active in CRPC [4]. Several different mechanisms have been demonstrated to drive AR-recurrence including: AR amplifications and overexpression, intratumoral testosterone synthesis, AR mutations, AR splice variants, aberrant regulation of the coactivators of AR, and others [4–9]. There is also increasing evidence that epigenetic modifications play a critical role in the development of CRPC. Specifically, alterations to the epigenetic state can provide a transcriptional landscape that allows the differentiated androgen-dependent epithelial cells to gain androgen-independent characteristics [10, 11]. It is only through the cellular stress of ADT that the cancer can overcome differentiation barriers through epigenetic alterations and adopt to castrate conditions [12]. Given this critical role, these epigenetic modifying proteins could offer promising pharmacological targets to treat CRPC.

Numerous lysine demethylases have also been shown to impact the growth and proliferation of PCa [13]. Metzger et al. demonstrated that KDM1A overexpression associates with poor clinical outcome in PCa [11]. KDM1A, an H3K4me1/2 demethylase, is proposed to change the substrate specificity of KDM1A toward H3K9me1/2 upon interaction with AR [11]. KDM3A interacts with AR through an LxxLL motif and regulates AR-target gene expression [14]. Demonstrating its importance in AR signaling, loss of KDM3A resulted in smaller testis size and decreased sperm count in mice [15]. While not linked to PCa, KDM3B is essential to spermatogenesis in vivo [16]. Similarly, KDM4A, KDM4B, and KDM5B can impact AR activity in PCa cells [17–19]. KDM4A, for example, interact with AR and overexpression of KDM4A leads to AR-mediated PSA transcription [17]. Clinical data suggests that KDM4A positively correlates with the aggressiveness of PCa tumors [20]. KDM4B can regulate AR recruitment and enhance transcriptional activity [18]. PHF8 (KDM7B) act as a coactivator of AR that is activated in hypoxic tumors [21, 22].

Much of this research has focused on the relationship between AR signaling and chromatin modifiers. Only a small number of epigenetic modifying enzymes have been studied in an androgen-independent context despite the extensive transcriptional alternations that occur during the development of ADT resistance. In stem cell biology, numerous epigenetic modifying enzymes have been identified that are required for cellular dedifferentiation to pluripotency [23, 24]. As both iPSC and CRPC progression involve cellular dedifferentiation, the genes involved in this process could potentially be similar. Therefore, in this work we systematically screened a focused shRNA library of chromatin modifying enzymes previously linked to stem cell differentiation to characterize their role in androgen-independent growth [23]. Our screen showed that perturbation in the histone methylation/demethylation homeostasis lead to alteration in the proliferative capacity of the cells. Among the hits that showed inhibitory effect, KDM3B was particularly interesting as it has not been studied in CRPC. We validated the specificity of the phenotype in CRPC cells and demonstrated that enzymatic activity of KDM3B is required. Knockdown of KDM3B led to changes in global gene expression associated with metabolic changes. To address the mechanism, we investigated metabolic changes under loss of KDM3B conditions. Overall, this work represents the first study to demonstrate the importance of KDM3B in CRPC.

## Results

### Knockdown of various chromatin modifiers alter proliferation in LNCaP-abl cells

**Fig. 1:**
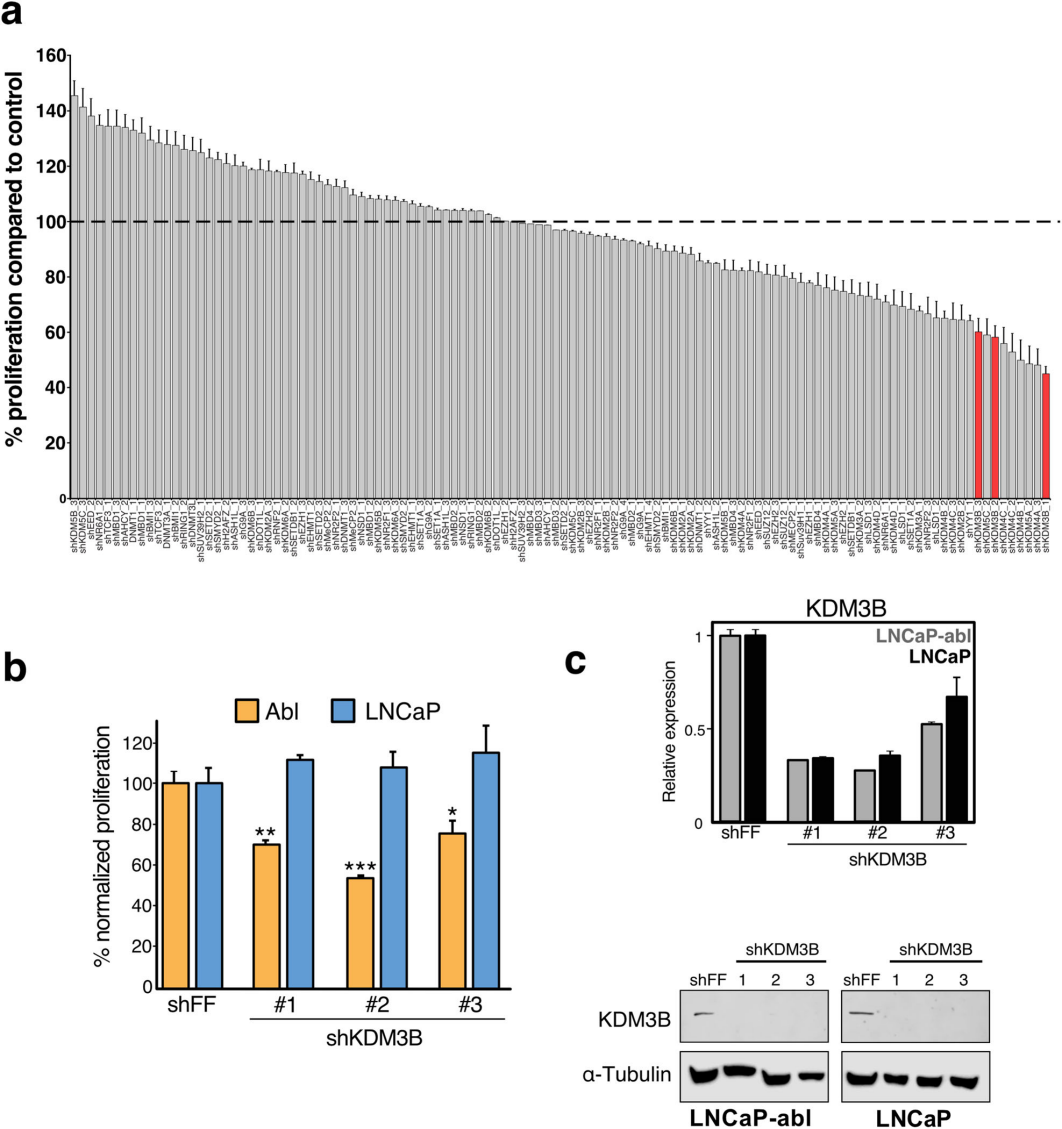
Knockdown of various chromatin modifying enzymes impacted proliferation of androgen-independent LNCaP-abl cells. **a** Cells were transduced with shRNA library targeting different enzymes. Following puromycin selection, cells were plated on a 96-well plate in equal numbers and then assayed for proliferation by MTS on day 7. Proliferation was normalized to a nontargeting shRNA control (shFF). Red bars showing shRNAs targeting KDM3B (*n* = 3, SEM). **b** LNCaP-abl and LNCaP WT cells were transduced with three independent shKDM3B and assayed as previously described. (*n* = 3, SEM, *p < 0.05, **p < 0.01, ***p < 0.001, statistically normalized to shFF control). **c** Relative KDM3B mRNA levels (top) and protein (bottom) following shRNA knockdown. RNA was normalized to ß-actin by 2^−ΔΔCt^ method (*n* = 3, SEM, *p < 0.05, **p < 0.01, ***p < 0.001). KDM3B protein expression levels are shown following treatment with three independent shRNAs in LNCaP-abl and LNCaP WT cells. ɑ-Tubulin was used as loading control.

To understand how hormone-sensitive PCa transition to CRPC, we conducted a focused shRNA screen of chromatin modifiers that had previously been shown to act as facilitators and barriers of somatic cell reprograming [23]. This includes chromatin modifying enzymes (methyltransferases, histone variant, methyl readers, and demethylases), transcriptional regulators, and E3-ubiquitin ligases (*n* = 119, targeting 47 different genes, Supplementary Table S1). This screen was done in LNCaP-abl cells, a model of androgen-independent CRPC derived from the androgen-sensitive LNCaP (Fig. 1a) [25] . From this screen, 43 individual shRNAs increased and 38 shRNAs decreased proliferation of LNCaP-abl. Approximately half of the hits with “proproliferative” effect (9/17 enzymes, average normalized proliferation of all shRNAs per target) were histone methyltransferases such as *EED* [26], and DNA methyltransferase 1 (*DNMT1*), both had been shown to correlate with PCa aggressiveness [26]. In contrast, 12 genes had an inhibitory effect on the growth of LNCaP-abl, including *KDM1A* and *EZH2*, which had previously demonstrated to be essential for CRPC growth [10, 11]. We also observed a cluster of JmjC-KDMs including KDM4A, KDM4B, and KDM5A that have been implicated for their role in PCa [27]. Overall, we found that most histone methylation associated enzymes and demethylases led to opposing effects, highlighting that histone methylation status is linked to cellular proliferation in CRPC.

**Fig. 2:**
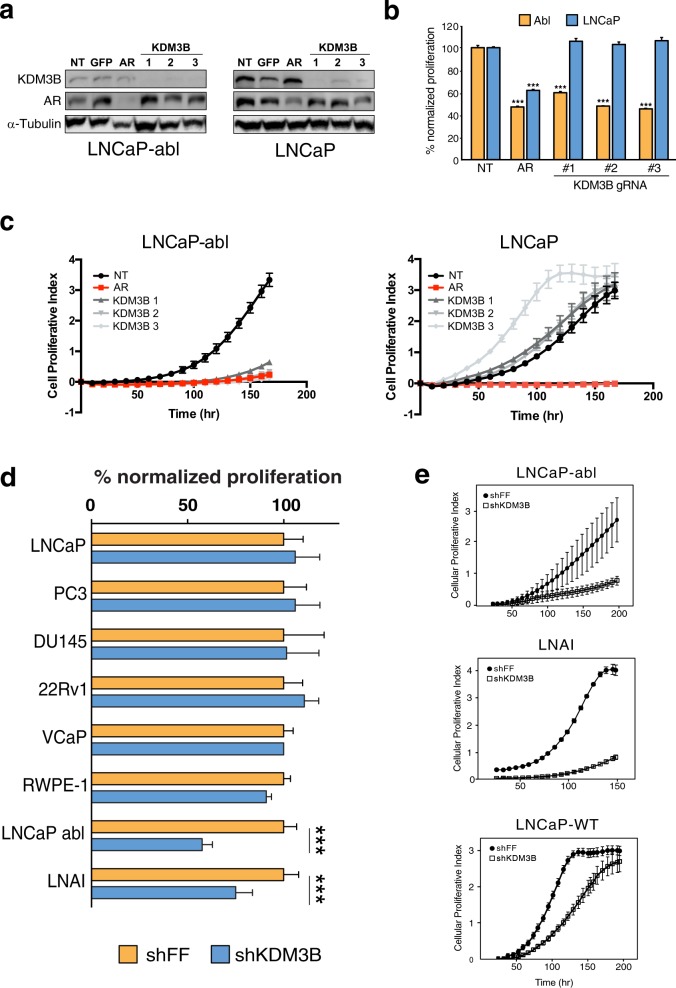
Downregulation or loss of KDM3B decreases the proliferation of androgen-independent cells. CRISPR/Cas9-mediated KDM3B knockout phenocopied the decreased proliferation phenotype in LNCaP-abl cells. **a** Western blot of LNCaP-abl Cas9 and LNCaP Cas9 cells transduced with targeted gRNA. The effect of KDM3B gRNA knockout on proliferation was assessed by both (**b**) MTS assay (*n* = 3, SEM, ***p < 0.001, statistically normalized to nontargeting control) and (**c**) RTCA. (*n* = 3, SEM, ***p < 0.001). **d** Knockdown of KDM3B caused decreased proliferation only in androgen independent cell lines. Cellular proliferation was measured by MTS (**d**) (*n* = 3, SEM, ***p < 0.001) and by RTCA (*n* = 3, SEM) (**e**) after cells were transduced, selected and grown for 1 week.

From our screen KDM3B knockdown was found to have the most significant antiproliferative effect in our CRPC model (Fig. 1a). To validate the screen results, we then retested the impact of KDM3B knockdown in both the parental androgen-dependent LNCaP and androgen-independent LNCaP-abl cells (Fig. 1b, Supplementary Table S1). Knockdown of KDM3B decreased the proliferation of LNCaP-abl cells but did not affect LNCaP cells. Efficient knockdown of KDM3B at the mRNA and protein levels was observed in both cell lines (Fig. 1c). Given the critical role of AR in LNCaP-abl, we next tested AR expression upon KDM3B knockdown. No effect on AR protein expression was observed (Supplementary Fig. S1a), suggesting that the observed phenotype was not due to altered AR expression. Supporting this, when we performed KDM3B knockdown in both castrate and androgen supplemented media we did not observe any change in the proliferation (Supplementary Fig. S1b). These results show that this lysine demethylase does not function through the AR signaling pathway. To confirm our loss-of-function results we generated CRISPR/Cas9 mediated KDM3B knockouts. Cells treated with Cas9 and three individual KDM3B or AR [28] targeting gRNAs showed targeted knockout of either AR or KDM3B (Fig. 2a). Consistent with our earlier results, knockout of KDM3B decreased the proliferation of LNCaP-abl but not LNCaP cells (Fig. 2b). To confirm that this phenotype was not specific to the assay used, we monitored the cell proliferation of LNCaP-abl and LNCaP using real-time cell analysis (RTCA). In this, the loss of KDM3B caused a significant decrease of LNCaP-abl proliferation (Fig. 2c). Similar to earlier results, knockout of KDM3B did not affect the growth of LNCaP. While one gRNA targeting KDM3B actually caused increased proliferation, this is likely an off-target effect as it was not observed with the other gRNA or shRNA.

Next, we investigated the effect of KDM3B knockdown in a variety of prostate cell lines. In these experiments, we used the most efficient shKDM3B (shKDM3B-1; #sh113). Knockdown of KDM3B did not alter the proliferation of RWPE-1 (immortalized prostatic epithelial), PC3 (AR negative), 22Rv1 (AR expressing and constitutively active splice variant), DU145 (AR negative), VCaP (AR expressing and constitutively active splice variant), and LNCaP [25, 29–35]. However, downregulation of KDM3B resulted in decreased proliferation of the two androgen-independent CRPC cells (LNCaP-abl and LNAI) (Fig. 2d). Knockdown of KDM3B was validated in all cells by qRT-PCR (Supplementary Fig. S2). To confirm these results, we tested the effect of shKDM3B on proliferation with LNCaP, LNCaP-abl, and LNAI cells by RTCA (Fig. 2e). Knockdown of KDM3B resulted in decreased proliferation of LNCaP-abl and LNAI, while LNCaP cells were less affected. Taken together, these results establish KDM3B as an important gene for the proliferation of AR-expressing androgen-independent CRPC cells.

### Loss of KDM3B causes decreased androgen-independent growth and the effect is dependent on KDM3B histone demethylation activity

**Fig. 3:**
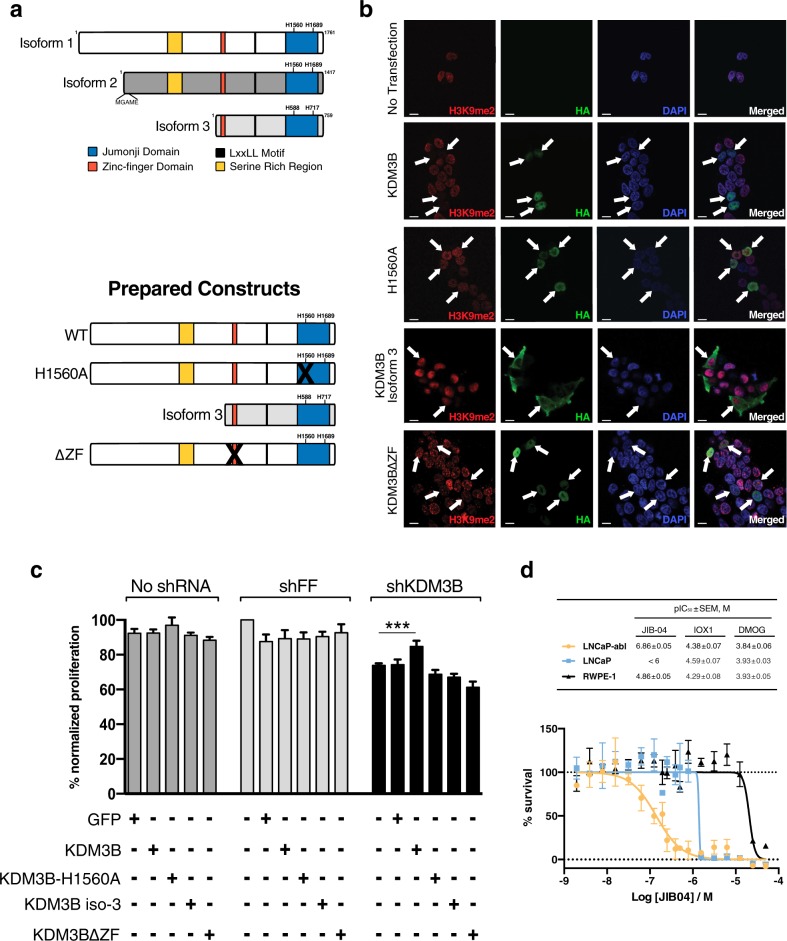
Histone demethylase activity of KDM3B is important for growth in LNCaP-abl cells. **a** Isoforms of KDM3B and cartoon representation of overexpression constructs. **b** Immunofluorescence microscopy of H3K9me2 following transfection with the overexpression construct. DAPI was used to stain nuclei. **c** Genetic rescue experiment of shRNA treated cells. Following transduction, cellular proliferation was assayed by MTS assay after 7 days (*n* = 13, SEM, ***p < 0.001, statistically normalized to shKDM3B control). **d** LNCaP-abl, LNCaP, and RWPE-1 cells were treated with small molecule inhibitors JIB-04, IOX1, and DMOG for 5 days. Cell viability was measured by MTS. Top: depicted pIC50 values for inhibitors were calculated by following equation pIC_50_ = −Log (IC_50_/M) for each cell line. Bottom: dose-response curve for JIB-04 treatment. (*n* = 2, SEM).

To characterize the role of KDM3B on androgen-independent growth in PCa, we conducted genetic rescue experiments in shKDM3B expressing cells. There are three reported isoforms of KDM3B (Fig. 3a). Isoform-1 (1761 AA) is proposed to be the canonical form with a Jumonji-C (JmjC) domain, C2HC4 zinc-finger domain, serine-rich domain, and a LxxLL motif [36, 37]. Isoform-2 is slightly shorter with a truncated N-terminus [38], while Isoform-3 lacks the first 1000 amino acids and does not contain the nuclear localization signal [38]. Isoform-2 is not commonly expressed and therefore was not included in our analysis [38–40]. In the KDM3B JmjC domain there are two histidine residues (His1560 and His1689) that interact with Fe(II) and are essential for demethylase activity [39]. In our rescue experiment we tested isoform 1 (wild type), isoform 3 (iso3), an enzymatically inactive JmjC domain (His1560Ala) and zinc-finger deleted (KDM3B∆ZF) KDM3B mutant (Fig. 3a). Each of these constructs had similar mRNA and protein expression compared with wild-type KDM3B (Supplementary Fig. S3). To demonstrate the enzymatic activity of these KDM3B constructs, we conducted immunofluorescence staining of H3K9me2 in transiently transfected cells. While all constructs were expressed, only the wild-type KDM3B was enzymatically active and could efficiently demethylated the H3K9me2 mark (Fig. 3b). Having validated these overexpression constructs, we then transfected them into LNCaP-abl cells treated with KDM3B shRNA. Expression of these rescue constructs did not affect the growth in cells treated with control shRNA (shFF). However, the enzymatically active KDM3B partially rescued the proliferation phenotype in the shKDM3B treated cells, while GFP (negative control), KDM3B-H1560A, iso3, and KDM3B∆ZF overexpression had no effect (Fig. 3c).

Next, we tested the effect of small molecule JmjC-inhibitors targeting the catalytic activity of KDMs on cell proliferation. We observed inhibition of the growth of LNCaP-abl (IC_50_ 0.14 µM) and LNCaP (IC_50_ ~1 µM) and a weaker inhibition in normal prostate epithelial cells RWPE-1 (IC_50_ ~20 µM) upon treatment with JIB-04, a pan-KDM inhibitor, which can also inhibit KDM3 (Fig. 3d) [41, 42]. Interestingly, androgen-independent LNCaP-abl was more sensitive than androgen-dependent LNCaP cells, in line with our shRNA studies. When we tested the broad spectrum 2OG oxygenase inhibitors, IOX1 [43] and DMOG (pro-drug of 2OG mimic N-oxalylglycine) [44], both PCa cell lines were found to be as sensitive as prostate epithelial RWPE-1 cells (Supplementary Fig. S4), suggesting KDM-independent mechanisms are also likely associated with these compounds. Overall, pan-KDM inhibition shows antiproliferative effect in PCa, particularly in LNCaP-abl CPRC cells.

Together with the RNAi data, our results suggest that the CRPC cell proliferation is, in part, dependent on the catalytic activity of KDM3B.

### KDM3B does not alter cell cycle or AR signaling in LNCaP-abl cells

**Fig. 4:**
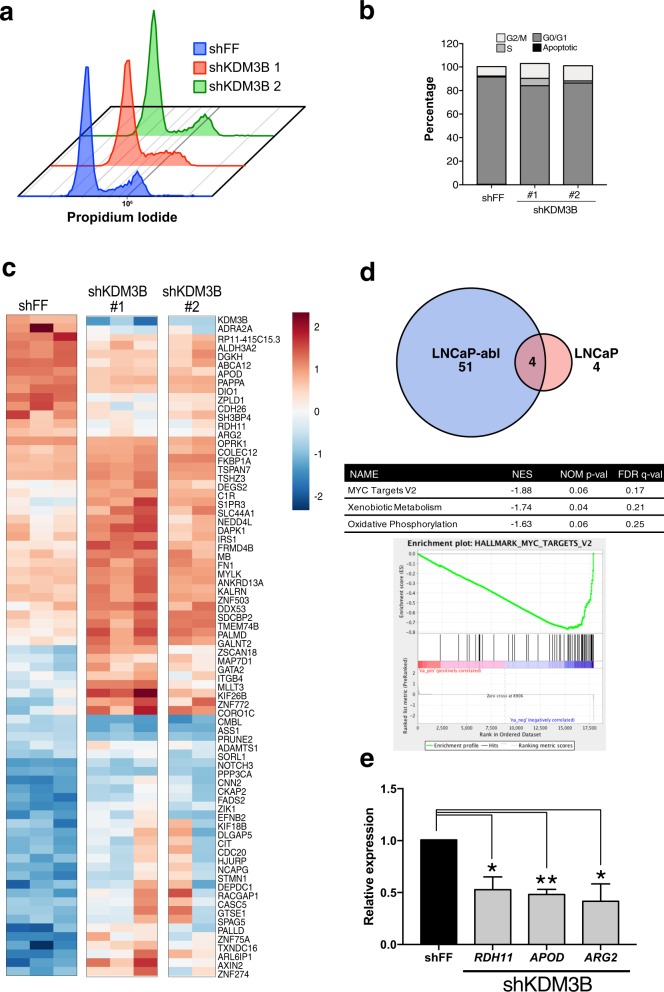
Knockdown of KDM3B did not cause a blockade in any cell cycle stage. **a** Overlay of PI staining in shFF, shKDM3B-1, and shKDM3B-2 treated LNCaP-abl cells. **b** Quantification of PI staining in the cells. KDM3B knockdown alters gene expression in LNCaP-abl. **c** Heatmap of differentially expressed genes (DEGs) with false discovery rate (FDR < 0.05) in LNCaP-abl. **d** Venn diagram of DEGs in LNCaP-abl and LNCaP and GSEA analysis performed with DEGs in LNCaP-abl. **e** Hits from DEGs with FDR < 0.05 were validated in shKDM3B-treated LNCaP-abl using qPCR (*n* = 3, SEM).

Having demonstrated that KDM3B is important to androgen-independent cell growth, we next investigated the molecular mechanism. We first assayed the cell cycle distribution after KDM3B knockdown. Interestingly, we did not observe any blockade at specific cell cycle stages (Fig. 4a, b, Supplementary Fig. S5). Although shKDM3B1 treatment exhibited slight alterations of S phase by PI staining this was not reproducible, and we did not observe the same phenotype with BrdU staining. Thus, KDM3B knockdown appears to cause a decrease in the cell cycle rate rather than an accumulation at a particular stage. We observed no significant difference in global H3K9me2 between shFF and shKDM3B suggesting that KDM3B may have locus specific targets in LNCaP-abl (Supplementary Fig. S6). To explore this, we characterized the transcriptome following KDM3B knockdown at two different time points based on our RTCA growth experiments, one at the lag phase (7 days post transduction) and another in the growth phase (14 days post transduction). At the early time point, a total of 149 genes were significantly differentially expressed (false discovery rate (FDR) <0.05) in LNCaP-abl cells treated with shKDM3B as compared with shFF. At the later time point (14 days post transduction), a total of 55 DEGs were found to be significant in shKDM3B relative to shFF. H3K9me3 is a critical repressive mark and is typically found in gene-poor regions leading to permanent repression [45]. However, in shKDM3B treated cells, the majority of differentially expressed genes (DEGs) were found to be upregulated, though the magnitude of the change in relative expression level was not dramatic. In contrast, almost no DEGs were found in shKDM3B treated LNCaP cells relative to shFF (Fig. 4c, d). Excluding *KDM3B*, only three DEGs were shared between LNCaP-abl and LNCaP cells: *ZIK1, ZSCAN18*, and *ARL6IP1*. In shKDM3B treated LNCaP-abl cells, commonly DEGs included: *KDM3B, ABCA12, PRUNE2, MAP7D1, APOD, ARG2, FN1, S1PR3, MYLK, CASC5, ARL6IP1, STMN1*, and *SDCBP2*. Gene set enrichment analysis indicated the enrichment of MYC targets in shKDM3B treated cells (Fig. 4d). In agreement with our earlier work, we did not observe any significant changes in AR regulated genes (Supplementary Fig. S1c). These results were validated with qRT-PCR (Fig. 4e, Supplementary Fig. S7). Of these, a number of metabolic genes including *ARG2* and *RDH11* were downregulated in shKDM3B cells (Fig. 4e). To investigate the clinical importance of our findings, we analyzed publicly available PCa expression data [46]. We did not detect any significant upregulation of KDM3B expression in CRPC patients as compared with primary PCa. Next, we compared the expression levels of three categories of KDM3B-regulated DEG in primary PCa versus CRPC [47] (Supplementary Fig. S8). We found that non-KDM3B associated genes (unchanged) were higher expressed in CRPC than primary PCa. This well-known phenomenon is proposed to be due to enhanced chromatin accessibility in CRPC [48, 49]. Compared with this, the DEGs impacted by KDM3B were expressed both higher (upregulated DEGs) and lower (downregulated DEGs) than non-KDM3B associated genes indicating greater KDM3B activity in CRPC. Overall these data suggest that KDM3B alters expression of both MYC targets and metabolic genes in LNCaP-abl cells and that these genes are elevated in late-stage PCa patients.

**Fig. 5:**
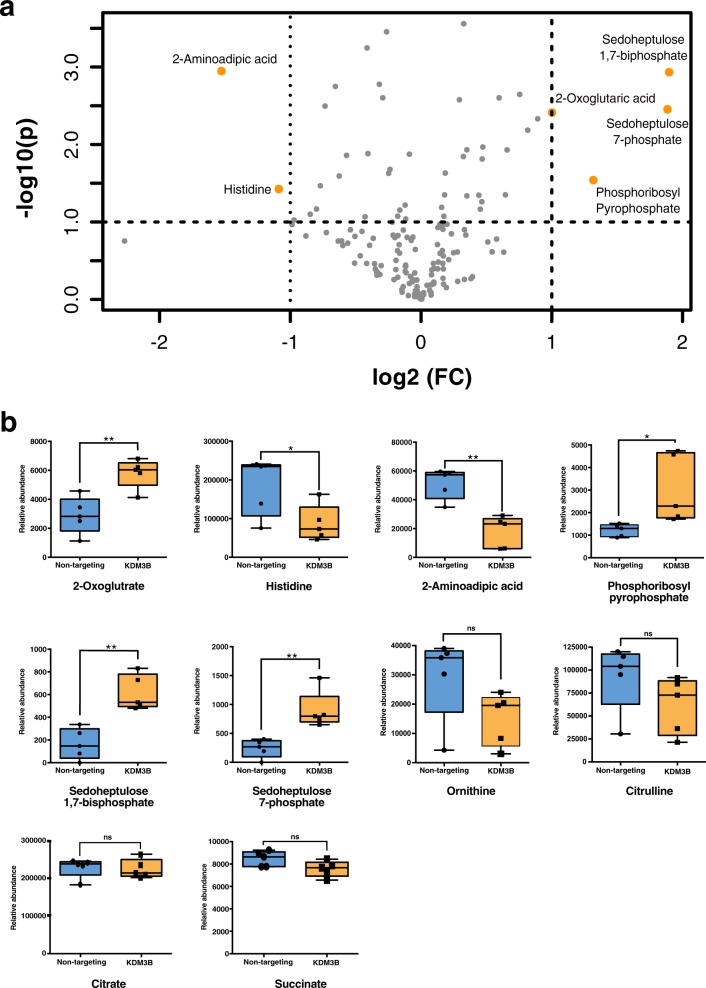
Untargeted metabolomic analysis of KDM3B knockout cells. **a** Volcano plot presenting all identified compound features (%CV < 30). Metabolites that were altered in the KDM3B knockout (fold change > 1.5 and FDR adjusted *p* value < 0.1) are highlighted in orange and annotated with the compound name. **b** Box plots showing the significance of differences in metabolites between KDM3B knockout (KDM3B) and nontargeting controls. (SEM, *n* = 5, FDR-adjusted * *p* < 0.1, **p < 0.05, ***p < 0.005).

Given that KDM3B knockdown alters metabolic gene expression but does not impact cell cycle, we proposed that the decrease in proliferation could be due to reduced cellular metabolic activity. To explore this, we examined the effect of KDM3B knockout on the relative abundance of metabolites in the cellular metabolome using untargeted liquid chromatography–tandem mass spectrometry (LC–MS/MS). Three separate LC–MS/MS methods were used to provide an overview of the cellular metabolome comparing gRNA-KDM3B1 treated cells with nontargeting gRNA treated samples (14 days post transduction; *n* = 5). Over 3000 compounds features were measured with a %CV < 30 but in general, few significant differences in metabolite feature abundances were observed. In total 170 metabolites were identified (matched to authentic standards, see “Materials and methods” section) of which six metabolites were significantly altered in abundance (fold change > 1.5, FDR adjusted *p* value < 0.1) following the KDM3B knockout (Fig. 5a) Interestingly, we observed a clear enrichment in 2-OG levels with the loss of KDM3B (Fig. 5b). As 2-OG is a cofactor of KDM3B and utilised during catalysis [36, 50], this observation is in agreement with its enzymatic mechanism. Other TCA metabolites such as citrate and succinate, (the latter being the by-product of KDM3B catalysis [36, 50]) remained largely unchanged (Fig. 5b). We also observed a marked, though nonsignificant, decrease in the arginase metabolite ornithine and downstream product citrulline (Fig. 5b). There was a clear enriched of the metabolites sedoheptulose-7-phosphate, sodeheptulose-1,7-phosphate, and phosphoribosyl pyrophosphate. Both 2-aminoadipate (found in the lysine degradation pathways [51]) and histidine were reduced in the KDM3B knockout.

## Discussion

Previous work has demonstrated that chromatin modifying enzymes play an important role in the cellular differentiation that occurs during CRPC progression [10, 11]. However, to date, a systematic large-scale study that characterizes the role of many different chromatin modifiers in CRPC is lacking. In this study, we conducted an unbiased loss-of-function screen targeting chromatin modifiers previously shown to be involved in stem cell development. We proposed that such enhancers or barriers of stem cell reprograming are likely to be involved in transition or maintenance of androgen-independent PCa [23]. Our screen suggested that the disruption of the chromatin methylation dramatically affects the proliferative capacity of the cells. Overall, knock down of methylation related enzymes appeared to enhance proliferation while loss of demethylases, in particular the H3K9 demethylases KDM4s/KDM3s, decreased growth in LNCaP-abl. Importantly, this was not due to shRNA off-target effects as a similar phenotype with both different shRNA and CRISPR/Cas9 was observed. KDM3B was only important in AR expressing androgen-independent CRPC cell lines but not androgen-dependent PCa or immortalized prostate epithelial cells. Our results suggest that KDM3B dependency is specific to a subset of CRPC cell lines that are used to model a clinically important patient population. As shown by experiments in androgen supplemented media and confirmed with the gene expression studies, the effect of KDM3B on CRPC proliferation was independent of AR signaling. In rescue experiments the phenotype was specific to the catalytic function of KDM3B. Only wild-type KDM3B was found to remove H3K9me2 and rescue the proliferative phenotype. Surprisingly, expression of ∆ZF KDM3B resulted in further decreased proliferation of shKDM3B treated cells. While speculative, this could be due to a dominant negative effect [52]. Biochemical studies demonstrated that zinc-finger domain and JmjC domain are required for KDM3A catalytic function due to homodimer formation via these domains [14, 53]. Overexpression of ∆ZF KDM3B may thus inactivate the endogenous KDM3B. Similar to KDM3A, KDM3B may undergo homodimerization; however additional studies are needed.

KDM3B is an H3K9me2 demethylase that belongs to the KDM3 family [39, 54]. Despite the contradictory results on the function of KDM3B, demethylation of H3K9me1/2 generally correlate with active transcription [40]. KDM3B is proposed to be a tumor suppressor gene in myeloid leukemia, colorectal cancer, and breast cancer [55–59]. It also contributes to repression of angiogenesis by downregulating ANGPT1 [38]. In contrast, studies also showed that KDM3B localizes to promoter region of *lmo2* gene and drives leukemogenesis [40]. A recent study demonstrated that knockout of KDM3B in mice led to defective spermatogenesis and litter size [16].

Despite the clear decrease in cellular growth of LNCaP-abl, the cell cycle distribution was not affected by KDM3B knockdown. While uncommon, this phenotype has been previously observed. Knockdown of 60S ribosomal proteins in human fibroblasts has been shown to result in decreased proliferation due to impaired ribosome biogenesis without changes in cell cycle distribution [60]. In MCF-10A cells, knockdown of the BRG1 or BRM1 subunit of SWI/SNF ATPase leads to decreased proliferation and in the absence of intact p53, cell cycle distribution remains unaffected [61]. Although KDM3B depletion does not cause cell death, it did significantly decrease proliferation in androgen-independent CRPC. This does not mean KDM3B is not clinically important. In our models of CRPC, the loss of KDM3B had a profound decrease on cellular proliferation.

Given the similar H3K9me2 levels detected in control versus shKDM3B, it is very likely that KDM3B possesses locus specific targets in androgen-independent CRPC. In accordance with this, analysis of the global gene expression changes by RNA-sequencing following KDM3B knockdown showed that a relatively limited number of genes were differentially expressed between the control and shKDM3B samples. Moreover, the DEGs were found to be elevated in clinical metastatic PCa [47]. The expression of AR or its target genes were unaffected by shKDM3B treatment, thus demonstrating that KDM3B, in contrast to other KDM members, does not act via AR [11, 18, 37, 62]. LNCaP cells were largely unaffected by KDM3B knockdown. However, we observed a clear decrease of *ARG2* and *RDH11* in LNCaP-abl cells. ARG2 is involved in arginase metabolism in the cells [63] and highly expressed in healthy prostate tissue [64, 65]. In agreement with these results we observed a marked decrease in the arginase metabolite ornithine. Interestingly, it has been reported that LNCaP cells cultured in androgen depleted medium led to decrease in ARG2 levels in an IL-8 dependent manner [63]. RDH11 is involved in the metabolism of retinoic acid and is highly expressed in prostate epithelium [66]. When cells are treated with all-trans retinoic acid (ATRA), RDH11 expression was found to decrease [67]. ATRA treatment in AML also leads to decrease in KDM3B levels as well [40]. Overall, these data suggest likely mechanisms in which CRPC growth is affected by KDM3B, which are independent of changes in canonical AR pathway or cell cycle distribution.

Metabolomic profiling revealed subtle changes in KDM3B knockout LNCaP-abl cells. The observed increase in 2-OG on KDM3B knockout likely reflect its reduced use as a cofactor in KDM3B catalysis [36]. However, several amino acids including histidine, tyrosine were reduced after KDM3B knockout. These have been demonstrated to be important in PCa. In an unbiased metabolomic analysis comparing blood samples from the patients with benign prostatic hyperplasia and PCa (Gleason score > 6), elevated levels of several metabolites including histidine, tyrosine, and others were found in PCa [68]. In the same study, these potential biomarkers were found to be associated with arginine metabolism and lysine degradation [68]. However, further investigation is required to better understand the role of KDM3B in metabolism.

While there are no selective KDM3 inhibitors to date, JmjC-KDM inhibitors show promise in PCa. The KDM4 inhibitor B3 demonstrate antiproliferative activity against both PCa and CRPC [69]. Pan-KDM inhibitor (targeting both KDM1 and KDM2-7 families) inhibit LNCaP cell proliferation, induce cell cycle arrest and apoptosis [70]. JIB-04, a pan-JmjC inhibitor that targets KDM2-7 including KDM3 [42, 71, 72], selectively inhibits growth of cancerous over normal prostate cells [41]. In this study, we have demonstrated CRPC LNCaP-abl are more sensitive to JIB-04 than LNCaP cells. While speculative, our results suggest that there are alterations in KDM dependencies between androgen-dependent and -independent PCa. Selective chemical tools for KDM3 subfamily, specifically for KDM3B, are required to decipher the mechanism of action and for target validation.

In conclusion, we have identified KDM3B to be important for the proliferation of CRPC cells. While the mechanism of action is complex, the requirement of the KDM3B catalytic activity for CRPC cellular proliferation suggests this may potentially be a novel pharmacological target.

## Materials and methods

### Plasmids, viral construction

The shRNA library was described previously [23, 73]. pENTR/TEV-D-TOPO-KDM3B plasmid was kindly gifted by Dr Catrine Johansson and Prof. Dr Udo Oppermann. This plasmid was used for cloning of three hemagglutinin tags to the C-terminal of KDM3B and transferred into gateway destination vectors. Primer sequences are given in Supplementary Table S2.

### Cell culture

LNCaP, PC3, 22Rv1, and DU-145 cells were cultured in RPMI1640 (Lonza, Switzerland) with 1% penicillin-streptomycin (Invitrogen, Netherlands) and 10% fetal bovine serum (FBS) (Gibco, Netherlands). LNCaP-abl and LNAI cells were maintained in RPMI 1640 (Lonza, Switzerland) with 1% penicillin-streptomycin (Invitrogen, Netherlands) and 10% charcoal stripped FBS (CSS) (Biowest, France). RWPE-1 cells were grown in keratinocyte serum-free media with required supplements (Gibco, Netherlands). HEK293T and VCaP cells were grown in DMEM (Lonza, Switzerland) supplemented with 10% FBS (Invitrogen, Netherlands) and 1% penicillin-streptomycin. All cells were grown in 37 °C humidified incubator with 5% CO_2_. LNCaP-abl cells were a generous gift from Dr Helmut Klocker. All cell lines used in this study were validated by short-tandem repeats profiling on July 24, 2016. Cells were routinely monitored for mycoplasma.

### Transfection of the cells and viral packaging

Cells were transfected according to the manufacturer’s instructions (FuGene, Promega, USA; Lipofectamine 3000-Thermo Fisher Scientific, USA). Viral packaging of plasmids was described previously [23].

### Transduction and stable cell line preparation

Cells were transduced with viral particles in the presence of 8 µg/ml protamine sulfate. Two days post infection, cells were selected with antibiotics and post selection, they were recovered for 1 day in their culture media. Finally, stable cells were expanded and used for further experiments.

### Short hairpin RNA knockdown

To generate KDM3B knockdown, cells were transduced with retrovirus particles containing shRNA. Knockdown was validated by qRT-PCR and/or western blot. shRNA sequences are given in Supplementary Table S1.

### gRNA-mediated knockout of KDM3B expression

CRISPR/Cas9 system was conducted to generate KDM3B knockout cells. Briefly, pLKO5.sgRNA.EFS.GFP plasmids (Addgene, #57822) [74] expressing single gRNA targeting KDM3B or AR was generated and transduced into cells stably expressing Cas9 protein. sgRNA sequences are given in Supplementary Table S2.

### Cell proliferation and cell cycle assays

For cell proliferation, cells (2000 cells/well) were plated into 96-well plates and MTS assay was performed as per the manufacturer’s instructions (Promega, USA). For RTCA experiment, cells (2500 cells/well) were plated into E-plate View (Acea Biosciences, USA) and measured every 20 min. For cell cycle analysis, shRNA treated cells (grown for 7 days) were stained with propidium iodide using a standard protocol [75]. For BrdU staining, shRNA treated LNCaP-abl cells (grown for 7 days) were treated with BrdU, washed, fixed, and DNA was denatured. Then, the cells were stained with anti-BrdU-FITC and propidium iodide and analyzed by flow cytometer. The staining experiments were repeated eight times, each time with knockdown validation. For inhibitor treatment, cells were treated with inhibitor for 5 days and viability was measured by MTS assay.

### Western blot

Protein lysates were extracted using lysis buffer supplemented with protease inhibitors, mixed with 4× Laemmli buffer (Bio-Rad, USA). Western blot was performed as previously described [76]. Antibodies are shown in Supplementary Table S3.

### Immunofluorescence staining

Transfected HEK293T cells were fixed with 1% paraformaldehyde, permeabilized using 0.5% Triton X-100, and blocked using 3% BSA. Primary antibodies were given overnight at 4 °C. Cells were then washed with PBS and incubated with secondary antibodies. Stained cells were placed on slides with Vectashield with DAPI (Vector Laboratories, USA) and visualized with Nikon Eclipse 90i at ×100 magnification.

### Gene expression analysis

For mRNA isolation, NucleoSpin^®^ RNA (Macherey-Nagel, Germany) kit was used as per the manufacturer’s instructions. cDNA was prepared busing MMLV-reverse transcriptase (Thermo Fisher Scientific, USA). qRT-PCR was performed with LightCycler^®^ 480 SYBR Green I Master (Roche, Switzerland). The primers used in qRT-PCR are listed in Supplementary Table S2.

### RNAseq

RNAseq experiment was performed at two different time points (*t* = 7 days and *t* = 14 days). For both time points RNA samples were sequenced on a HiSeq4000, the reads were mapped on hg19 genome build by HISAT2 software. mRNA counts were calculated using HTSeq software. DEGs were identified using DESEQ2. Gene set enrichment analysis was performed by using publicly available gene sets data (http://software.broadinstitute.org/gsea/msigdb/index.jsp) [77]. Gene Expression Omnibus accession number for RNAseq analyses is GSE127904.

### KDM3B DEG expression in CRPC patients

Based on their response to shKDM3B, DEGs were grouped into downregulated, unchanged, and upregulated. RPKM values for DEGs in primary and metastatic PCa samples were obtained from cBioPortal (GSE21032) [47, 78, 79]. Wilcoxon test was used for statistical analysis.

### Metabolic profiling

Metabolites from LNCaP-abl cells treated with gRNAs for KDM3B knock out (*n* = 5) were extracted into ice-cold methanol. Cells lysate was collected, centrifuged, DNA content was measured and filtered (10 kDa MWCO). The samples were analysed using three LC-MS methods: anion-exchange chromatography-mass spectrometry and reversed-phase chromatography-mass spectrometry (positive and negative ion mode). Post-sample analysis, Progenesis QI software, was used for raw data processing, which included peak alignment, isotope cluster recognition, and compound identification. Compound identification was performed by matching compound features to four measurement parameters associated with authentic standards. Matches were based on accurate mass measurement (<5 ppm), experimental retention window time (<30 s), matching fragmentation patterns where applicable and isotope patterns matched with the standard (>90%). MetaboAnalyst was used for data processing and statistical analysis. FDR adjusted *p* values were used throughout for analysis of metabolomics data (Fig. 5) to account for the size of abundance differences in comparison to the overall variance (i.e., the range of abundance values).

### Statistical analysis

Statistical analysis is described in the figure legends where *n* describes number of biological replicates. All in vitro experiments were performed at least three independent times (except Fig. 3d and Supplementary Fig. S4). Mean ± S.E.M. was used for data representation. Two tailed Student’s *t* test was used for the comparison of the difference between two groups. For RNAseq, FDR < 0.05 was used as significance cutoff. In all figures (except Fig. 5), *p < 0.05, **p < 0.01, and ***p < 0.001 was used to demonstrate significance.

## Supplementary information

Supplementary Table S1

Supplementary Table S2

Supplementary Table S3

Supplementary Figure Legends

Supplementary Figure S1

Supplementary Figure S2

Supplementary Figure S3

Supplementary Figure S4

Supplementary Figure S5

Supplementary Figure S6

Supplementary Figure S7

Supplementary Figure S8
