# Supplementary material for: Systematic characterization of chromatin modifying enzymes identifies KDM3B as a critical regulator in castration resistant prostate cancer
Source: Oncogene. 2019 Dec 10;39(10):2187–201. doi: 10.1038/s41388-019-1116-8 (PMC7056651; doi:10.1038/s41388-019-1116-8)
Supplement: Supplementary file 4 — Supplementary Figure Legends [file 41388_2019_1116_MOESM4_ESM.docx]

**SUPPLEMENTARY FIGURE LEGENDS**

**Supplementary Figure S1.** KDM3B knockdown does not affect canonical AR signaling. **(a)** AR protein levels upon shKDM3B treatment in LNCaP-abl cells. **(b)** The effect of shKDM3B treatment in charcoal stripped serum and androgen supplemented serum (1 nM Testosterone) on LNCaP-abl and LNCaP cells (n=3, SEM) **(c)** Heatmap of important AR signaling members are shown in shFF- or shKDM3B-treated LNCaP-abl.

**Supplementary Figure S2.** KDM3B knockdown was validated by qRT-PCR. KDM3B mRNA levels were normalized to β-actin and calculated by 2^-ΔΔCt^ method (n=3, **p<0.01, ***p<0.001).

**Supplementary Figure S3.** Validation of expression of KDM3B overexpression constructs (isoform-1, His1560Ala mutant, isoform-3 and ΔZF) at mRNA and protein level in HEK293T cells.

**Supplementary Figure S4.** Dose-response curves for IOX1 and DMOG treated LNCaP-abl, LNCaP and RWPE-1 cells. The treatment was done for 5 days and cell viability was measured by MTS (n=2, SEM).

**Supplementary Figure S5.** BrDU Staining showed no difference between (**a)** shFF and **(b)** shKDM3B-1 treated LNCaP-abl cells. The cells were transduced, selected with puromycin. 7 days later, they were fixed and stained with Alexa-488 Conjugated BrDU and PI. **(c)** Overlay of BrDU-PI stained shFF and shKDM3B-1 treated LNCaP-abl.

**Supplementary Figure S6.** Comparison of global H3K9me2 levels in shFF or shKDM3B1 treated LNCaP-abl cells.

**Supplementary Figure S7.** qRTPCR validation of DEG in LNCaP-abl cells (n=3, *p<0.05, **p<0.01).

**Supplementary Figure S8:** Altered differentially expressed genes (DEG) expression in primary and metastatic prostate cancer patients (GSE21032). DEGs were divided into three groups based on their response (log-fold change) to shKDM3B treatment in LNCaP-abl cells: Upregulated: log fold change > 0.5; downregulated: log fold change < -0.5; unchanged: -0.5 < log fold change < 0.5.
