# Supplementary figures and images for: Systematic characterization of chromatin modifying enzymes identifies KDM3B as a critical regulator in castration resistant prostate cancer
Source: Oncogene. 2019 Dec 10;39(10):2187–201. doi: 10.1038/s41388-019-1116-8 (PMC7056651; doi:10.1038/s41388-019-1116-8)

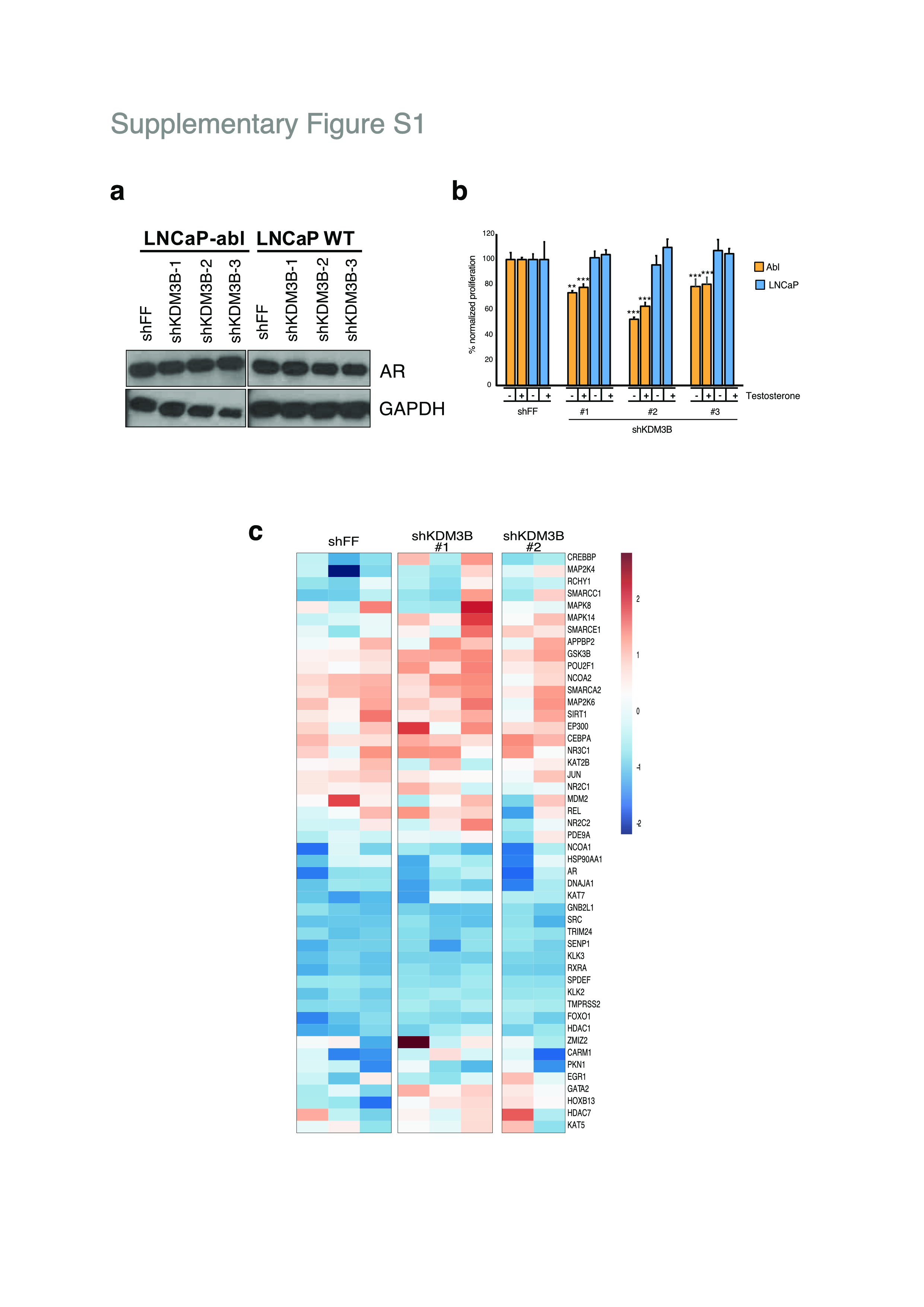

Supplement: Supplementary file 5 — Supplementary Figure S1 [file 41388_2019_1116_MOESM5_ESM.tif]

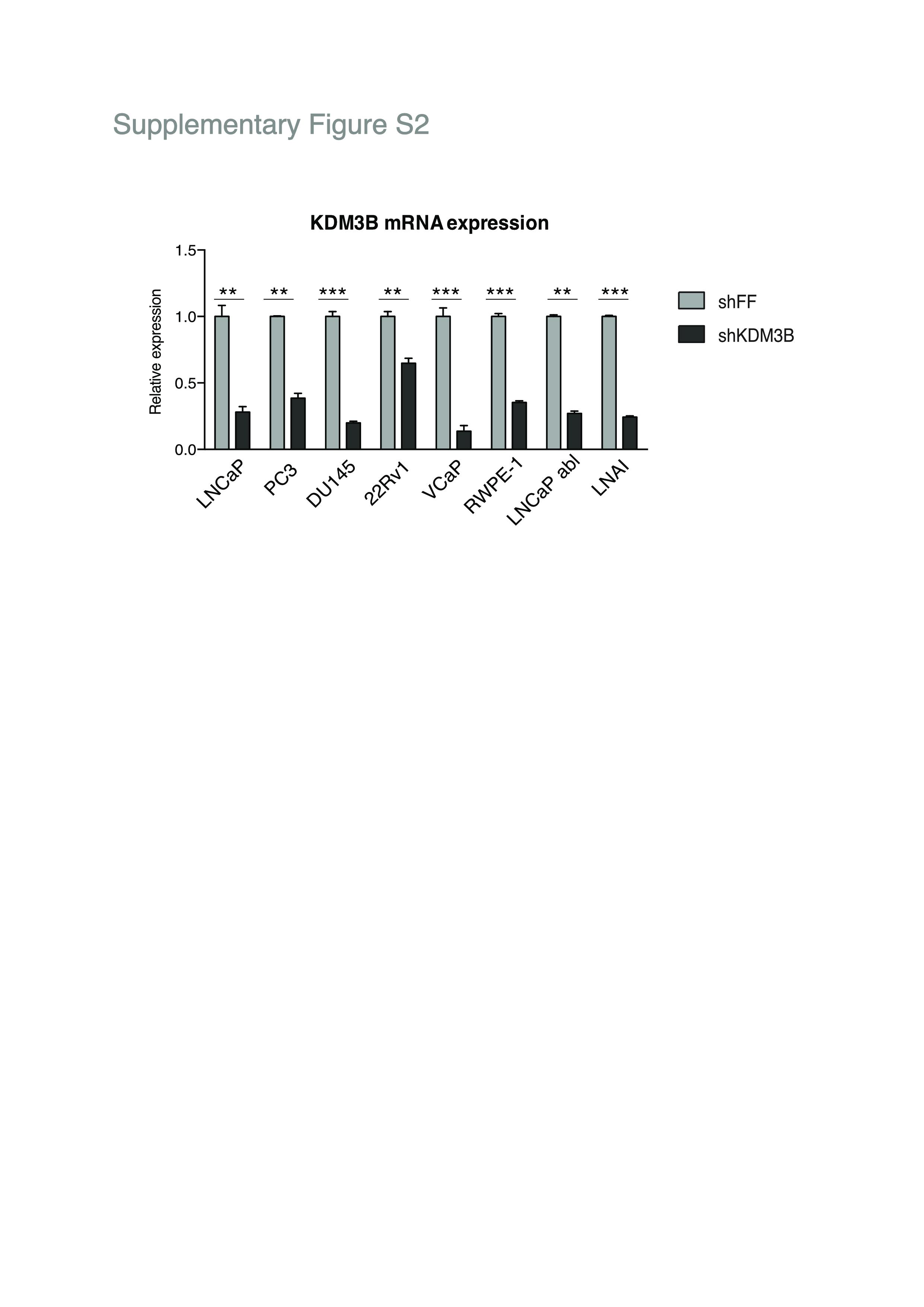

Supplement: Supplementary file 6 — Supplementary Figure S2 [file 41388_2019_1116_MOESM6_ESM.tif]

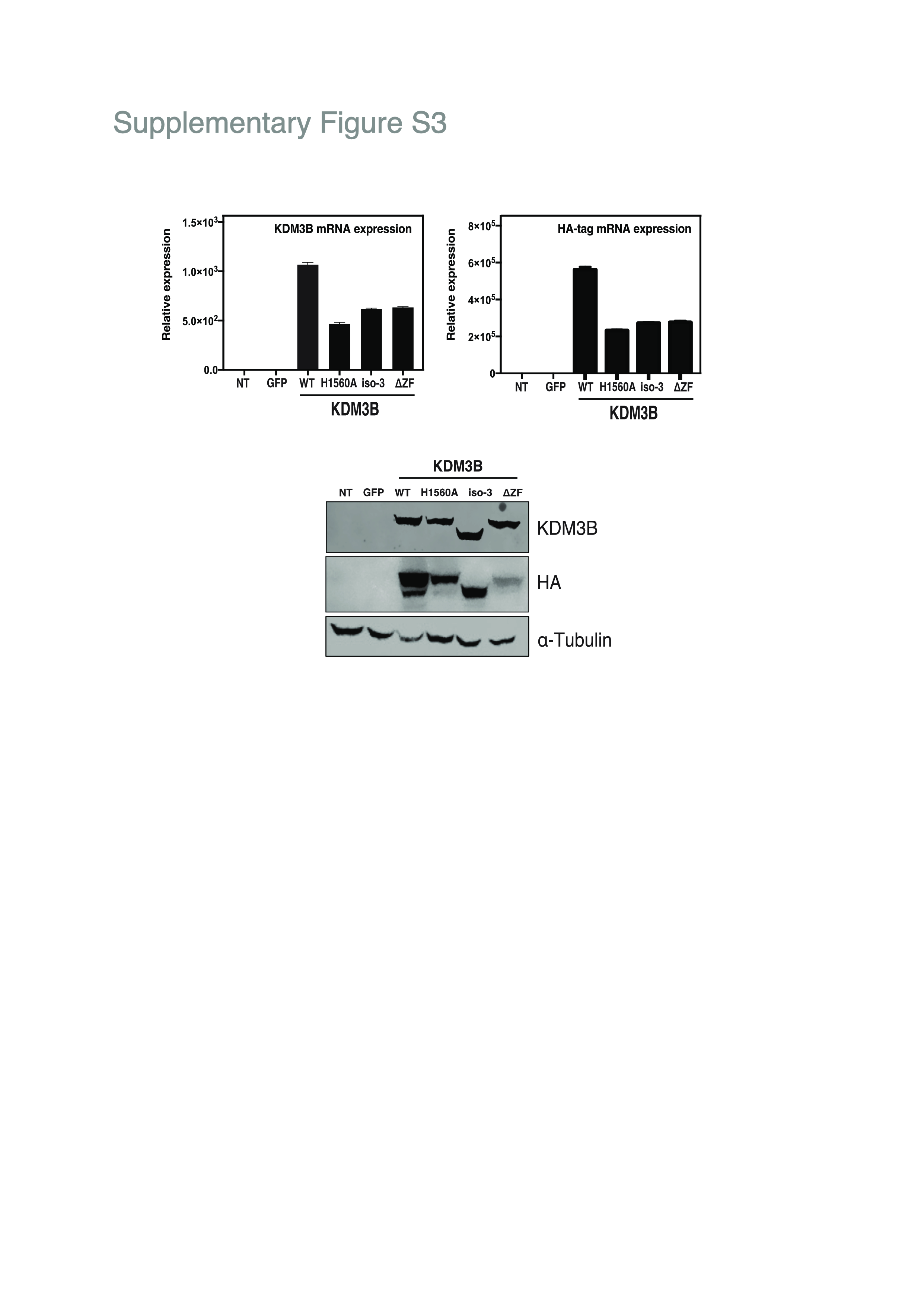

Supplement: Supplementary file 7 — Supplementary Figure S3 [file 41388_2019_1116_MOESM7_ESM.tif]

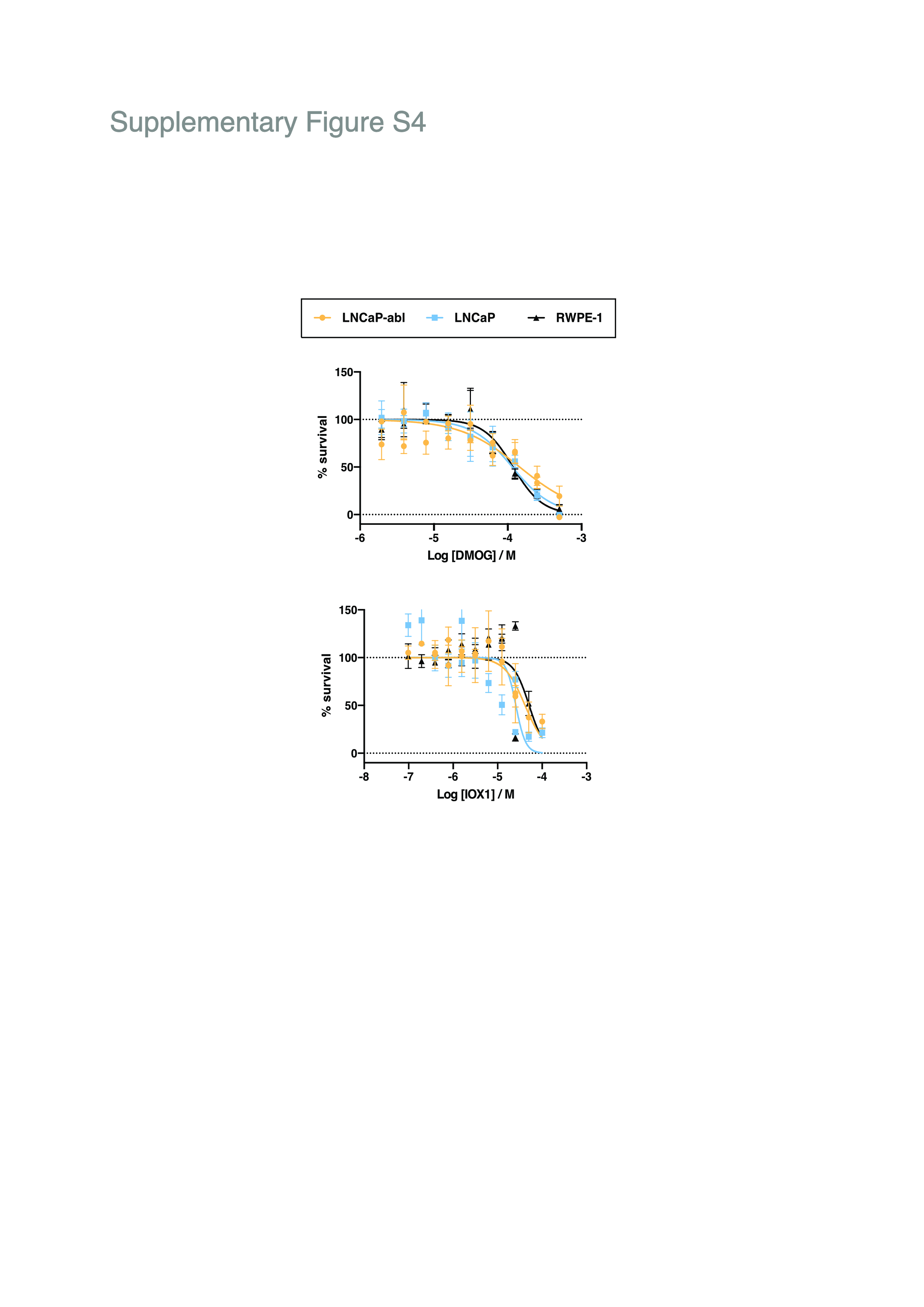

Supplement: Supplementary file 8 — Supplementary Figure S4 [file 41388_2019_1116_MOESM8_ESM.tif]

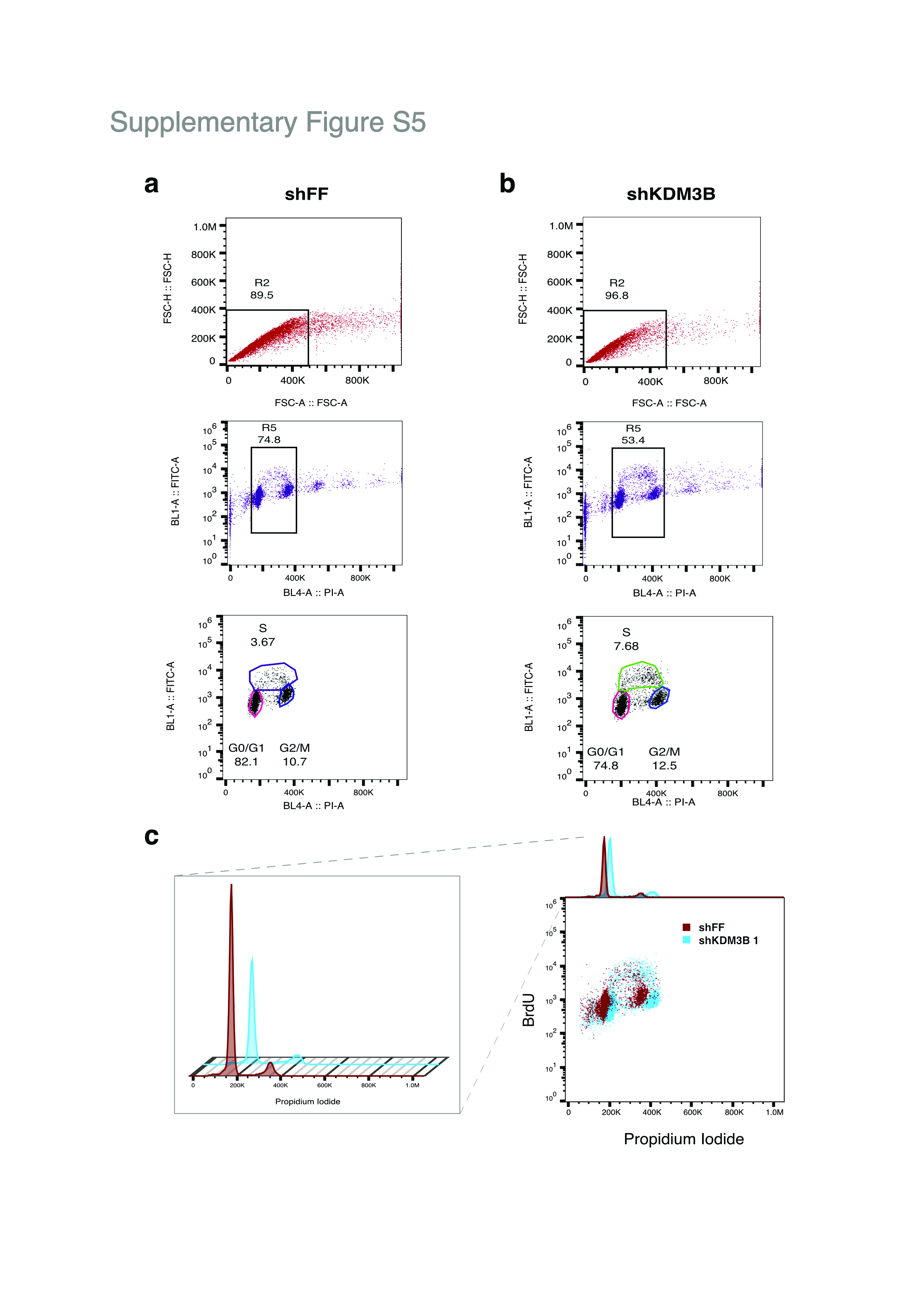

Supplement: Supplementary file 9 — Supplementary Figure S5 [file 41388_2019_1116_MOESM9_ESM.tif]

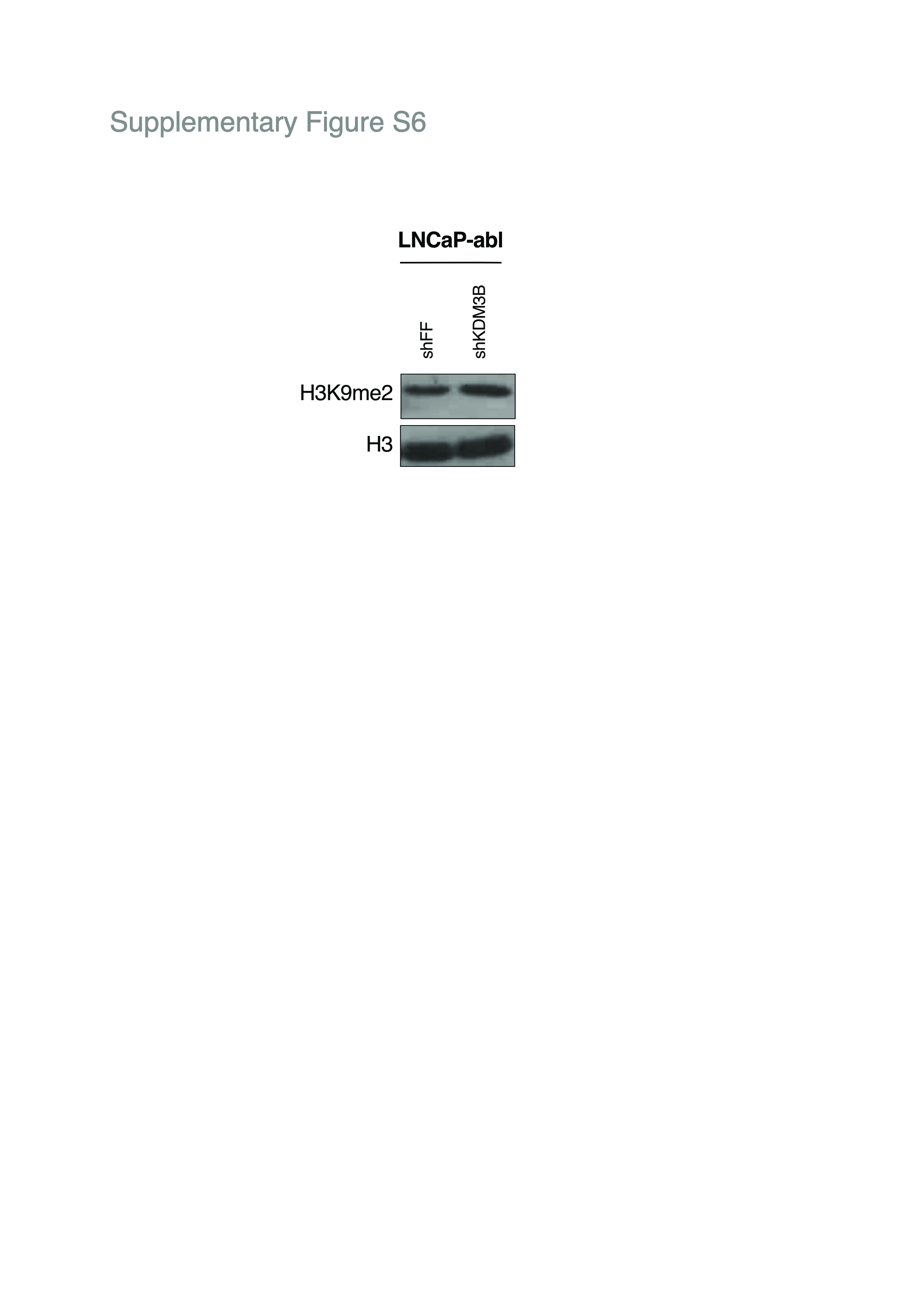

Supplement: Supplementary file 10 — Supplementary Figure S6 [file 41388_2019_1116_MOESM10_ESM.tif]

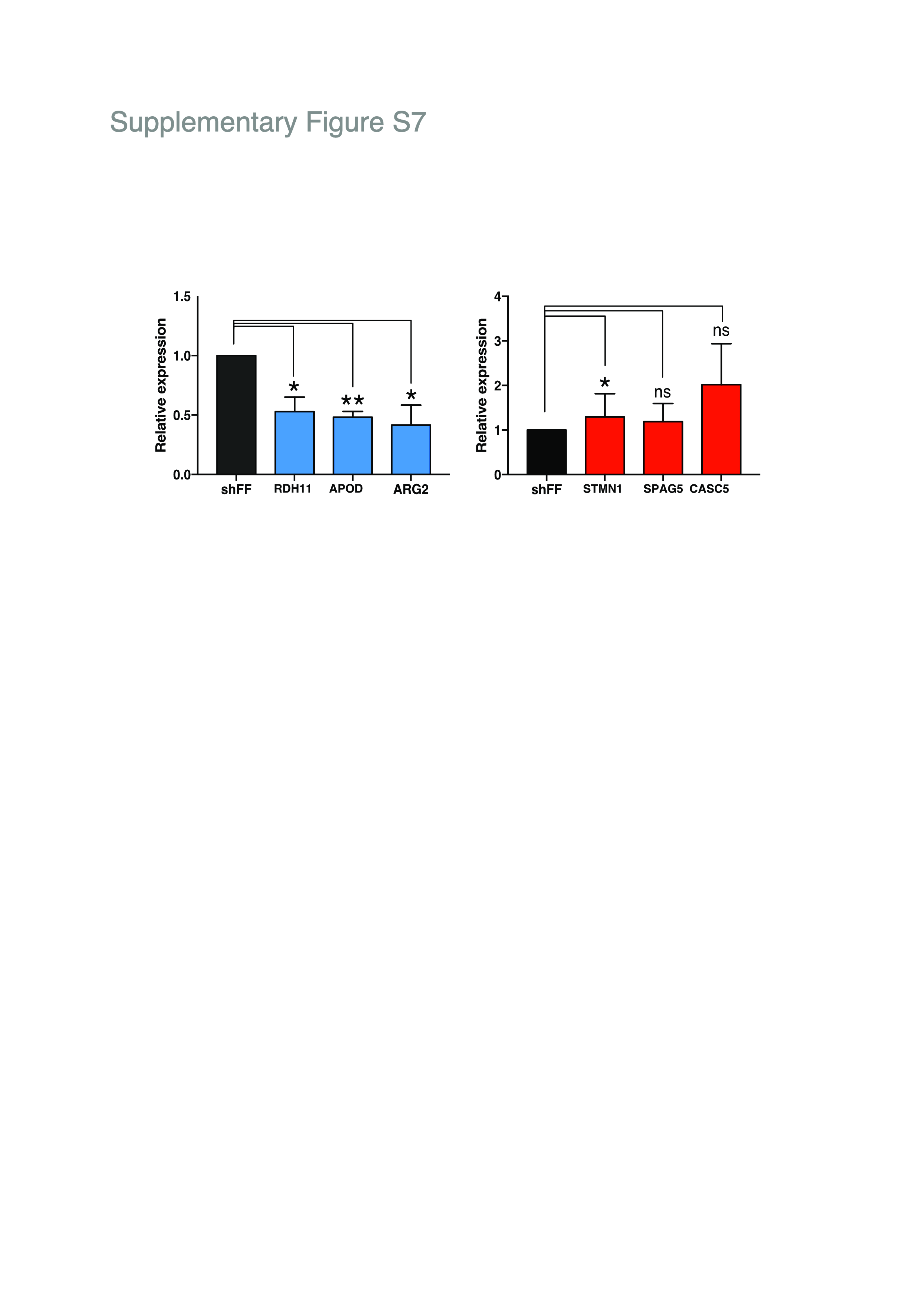

Supplement: Supplementary file 11 — Supplementary Figure S7 [file 41388_2019_1116_MOESM11_ESM.tif]

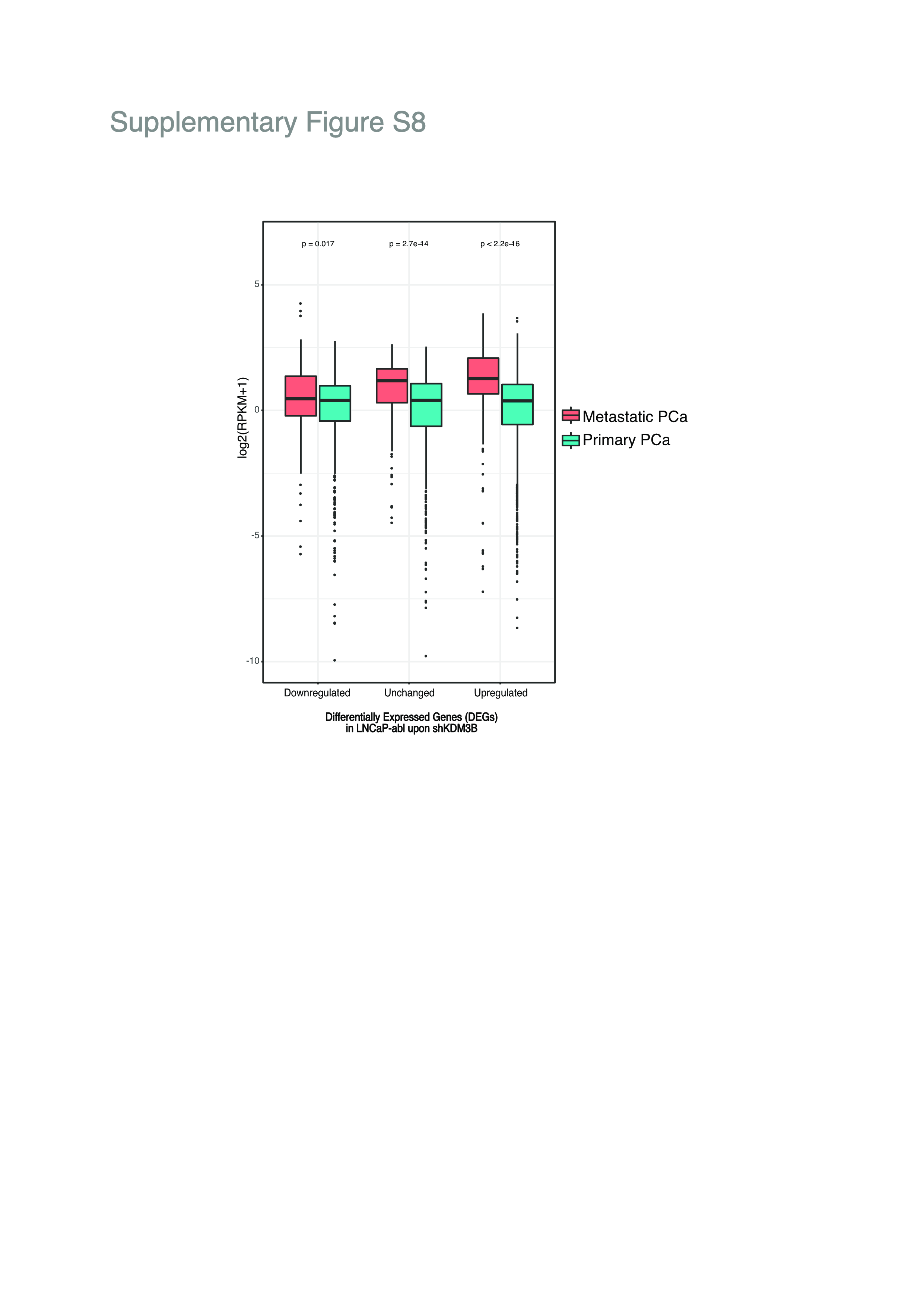

Supplement: Supplementary file 12 — Supplementary Figure S8 [file 41388_2019_1116_MOESM12_ESM.tif]
